# Differential expression of Homeobox C11 protein in water buffalo *Bubalus bubalis* and its putative 3D structure

**DOI:** 10.1186/1471-2164-15-638

**Published:** 2014-07-30

**Authors:** Monal Sharma, Leena Rawal, Deepak Panwar, Neeta Sehgal, Sher Ali

**Affiliations:** Molecular Genetics Laboratory, National Institute of Immunology, Aruna Asaf Ali Marg, New Delhi, 110067 India; Department of Zoology, University of Delhi, Delhi, 110007 India

**Keywords:** HOXC11 protein, Water buffalo, Gene expression, Protein characterization, Protein modeling

## Abstract

**Background:**

The Homeobox (*Hox)* family complex contains 39 genes, clustered into four groups (A-D) all expressing in sequential manner. The HOX proteins are transcriptional factors involved in regulation of pattern formation of the anterio-posterior body axis across the species. Most of the *Hox* family genes have been studied with respect to their organization and expression during the embryonic stages. However, expression pattern of Homeobox C11 (*Hoxc11*) gene in the 5′ region, particularly in higher mammals remains largely unexplored.

**Results:**

We cloned and expressed Homeobox C11 (*Hoxc11*) gene from water buffalo *Bubalus bubalis.* The recombinant HOXC11 protein expressed as inclusion bodies was solubilized in Tris buffer (10 mM, pH-6.5) and purified using Ni-NTA affinity column. The purity and molecular weight of HOXC11 protein (~33 kDa) were confirmed by SDS-PAGE and western blot analysis. Employing immunohistochemistry approach, we localized HOXC11 protein in the nuclei across the tissues of buffalo. Western blot analysis showed highest expression of HOXC11 protein in kidney and lung although its possible renal and respiratory roles are not yet established. Electrophoretic mobility shift assay (EMSA) demonstrated the specific binding of HOXC11 protein with the promoter element, CE-LPH1 of lactase-phlorizin hydrolase (*LPH*) gene showing reduced mobility of the protein-DNA complex, corroborating with earlier report on the possible role of this protein in intestinal functions. *In silico* analysis of HOXC11 showed predominance of α helices and presence of six conserved domains. We deduced the putative 3D structure of HOXC11 protein and fifteen possible DNA interacting residues within the homeodomain.

**Conclusions:**

Present study augments our understanding on the specific expression of HOXC11 protein in kidney and lung in water buffalo. The fifteen DNA interacting residues reported herein provide an opportunity to establish much broader structural and functional perspectives of HOXC11 protein in the context of genome analysis in general and animal biotechnology in particular.

**Electronic supplementary material:**

The online version of this article (doi:10.1186/1471-2164-15-638) contains supplementary material, which is available to authorized users.

## Background

Gene expression within the realm of time and space is a hallmark ensuring the morphogenesis, development and sustenance of an organism. Based on tissue specific expression profile, often structure-function relationship is established. A set of genes reportedly involved in regulating shape and orientation during the early embryonic development are assisted by *Hox* family genes [1]. The *Hox* family genes have been characterized in the context of pattern formation from *Drosophila* to *Homo sapiens*. In human, 39 *Hox* genes organized into four distinct clusters (A, B, C and D) are located on chromosomes 7, 17, 12 and 2, respectively [2]. Each cluster in turn is composed of 9–13 closely related genes giving rise to distinct paralogs and often, although not always, have overlapping functions. These paralogs arranged in a collinear manner are successively activated, from ‘head’ to ‘tail’ orchestrating sequential expression thus, regulating development [3, 4] *.*

*Hox* genes from insect to model vertebrate species have been studied to explore its organizational and functional status during embryonic development [5]. In the mouse embryo, *Hoxc11* mRNA expression has been observed predominantly in posterior regions especially in the hind limbs, kidney and developing genitalia [6]. Of all the *Hox* family genes studied thus far, due attention has not be given to the members of 5′ region, particularly *Hoxc11*.

The *Hox* genes encode transcription factors that contain a segment of conserved polypeptide designated as homeodomain which controls the formation of anterioposterior (AP) body axis [7, 8]. Most of the homeodomains are 60 amino acids in length (although exceptions are known). The homeodomain makes major groove contact, via helix turn helix motif, and minor groove contacts, via the N-terminal arm of the homeodomain with DNA [9]. Studies have demonstrated that *Hox* genes harbor overlapping domains of expression in the developing embryos [10]. Only a few homeotic response elements (HOMREs) have been characterized at the molecular level [11]. The presence of multiple binding sites in HOMRE can influence DNA-binding specificity by facilitating cooperative homeotic protein interactions. The identification of the functional domains of *Hox* gene products depends on the physiological context in which the HOX protein interacts with the target DNA. HOX proteins have been demonstrated to act as positive or negative regulators of the transcriptional activity of very specific target in the cultured embryos [12]. Consequently, HOX proteins play a crucial role in maintaining cell differentiation and proliferation across the species [13–16]. Their expression has been observed in oocytes and early embryos of human, mouse, porcine and bovine [17–19]. However, expression of HOXC11 protein in adult animals still remains a subject of investigation. Notwithstanding such information available on this line, strange it may seem, no attempt has been made to uncover possible interacting residues within the homeodomain of HOXC11 protein. Similarly, no information is available on the distribution of this protein within the cell systems in adult animals.

Buffalo is an economically important livestock species in the Indian sub-continent and South Asian countries [20]. We characterized HOXC11 protein from water buffalo *Bubalus bubalis* focusing particularly on its tissue specific expression, demonstrating its localization in the nuclei and finally deducing its putative 3D structure. Prospects of the present study in the context of biological relevance of HOXC11 protein are highlighted.

## Results

### Expressional validation of HOXC11 protein

**Figure 1:**
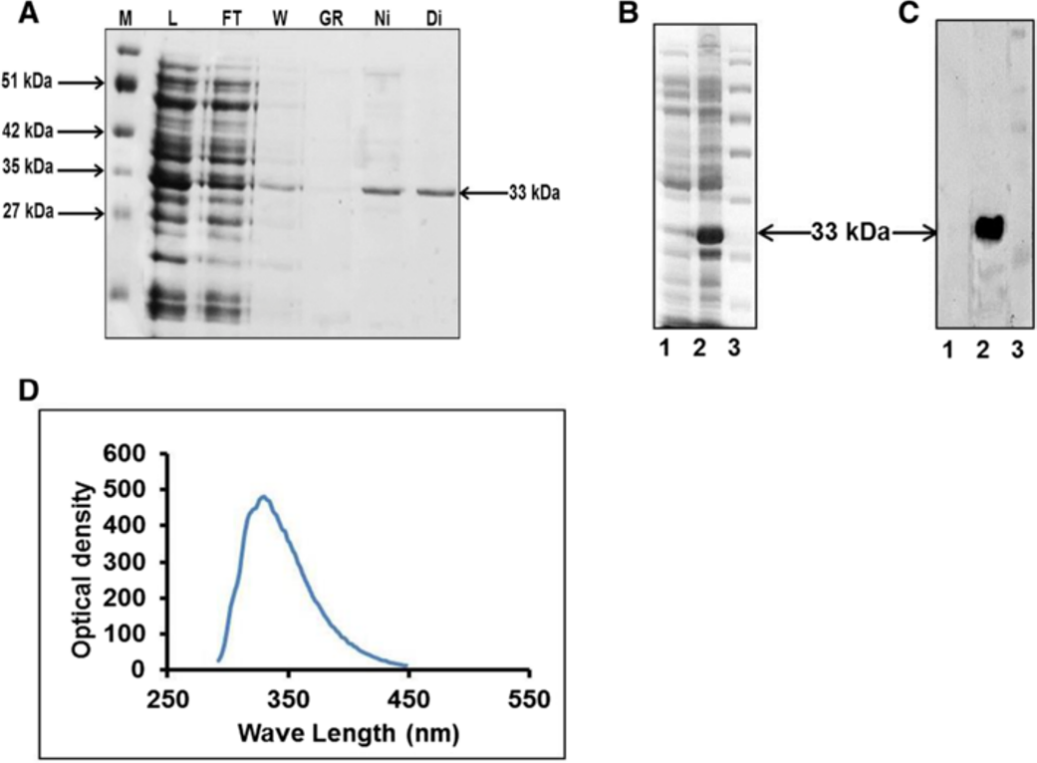
**SDS-PAGE profile of buffalo HOXC11 protein. A)** SDS-PAGE (15%) for HOXC11 protein showing its resolved chromatographic fractions. M denotes marker, L: load, FT: flow through, W: wash, Gr: gradient, Ni: Ni-NTA fraction and Di: dialyzed protein. **B)** SDS-PAGE of HOXC11 protein; lane 1 represents uninduced; lane 2, induced and lane 3, marker. **C)** Validation of purified buffalo HOXC11 protein by Western blot with anti-His and HRP conjugated anti-rabbit IgGs used as primary and secondary antibodies, respectively. Lane 1, represents uninduced; lane 2, induced and lane 3, marker. **D)** Tryptophan emission spectra. Fluorescence of refolded protein was scanned at 280 nm excitation wavelength and 300–400 nm emission wavelength.

**Figure 2:**
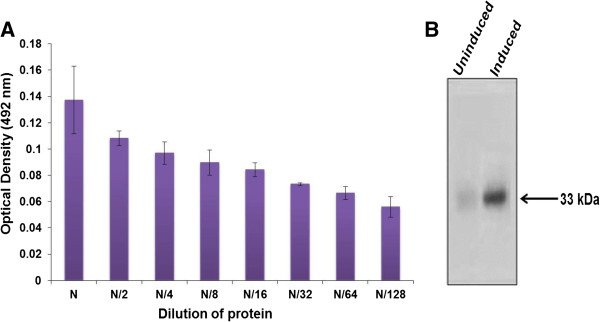
**Immunoreactivity of recombinant HOXC11 protein. A)** ELISA: Immunoreactivity of refolded HOXC11 was analyzed by primary antibody (rabbit anti-HOXC11) and secondary antibody (HRP labelled goat anti-rabbit IgG). Optical density was taken at 492 nm. N indicates neat protein and N/2 to N/128 represent the different dilutions of HOXC11 protein. **B)** Western blot for purified protein using specific antibodies against HOXC11 protein. Note the 33 kDa band in the induced sample confirming the purified protein.

Recombinant HOXC11 protein expression was induced with 1 mM IPTG at 37°C for 4 hours. The recombinant protein expressed in *E. coli* as inclusion bodies was solubilized in Tris buffer (10 mM, pH-6.5) and purified to homogeneity by immobilized metal chromatography (Figure 1A). The SDS-PAGE and western blot analysis showed a purified HOXC11 band of approximately 33 kDa, corresponding to theoretical molecular weight of protein (Figure 1B and C). UV fluorescence spectra showed a maxima at 330 nm evidencing HOXC11 protein has buried tryptophan residues in the non-polar environment (Figure 1D). MALDI-TOF/TOF determined the identity of HOXC11 protein and detected peaks were matched against Mascot peptide mass fingerprint database for homology search (Additional file 1). ELISA and western blot analysis reflected the immuno-reactivity and purity of HOXC11 protein (Figure 2A and B).

### HOXC11 secondary structure

**Figure 3:**
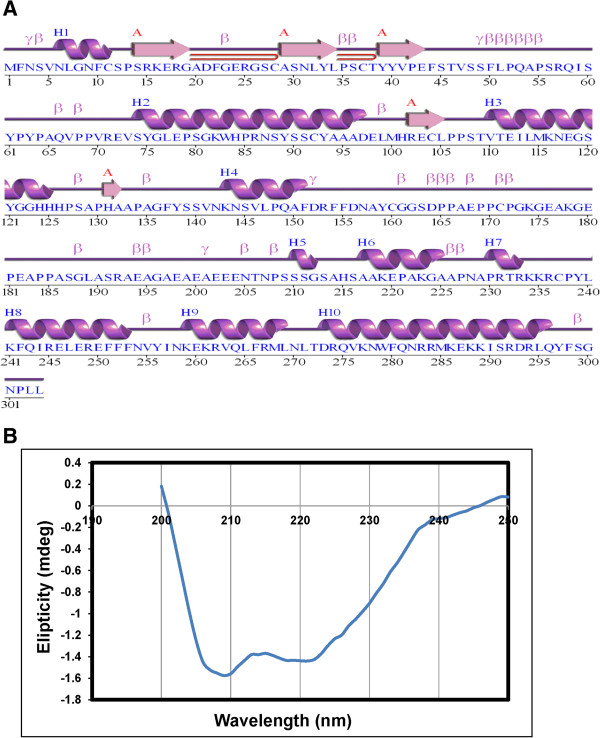
**Secondary structure of HOXC11 protein. A)** Profunc predicted secondary structure of HOXC11 protein. The amino acid sequence is capitalized. H1-H10, β, γ and  represent helices, beta turn, gamma turn and beta hairpin, respectively. **B)** CD spectra of refolded HOXC11 protein. Peaks at 208 nm and 222 nm indicate presence of α helices.

The secondary structure of buffalo HOXC11 protein predicted by ProFunc server revealed the presence of single sheet, two beta alpha beta units, two beta bulges, five strands, ten helices, seven helix-helix interacts, thirty one beta turns and eight gamma turns (Figure 3A). Further, to validate our observation, we performed far-UV circular dichroism spectroscopic analysis for HOXC11 protein. The CD spectrum of refolded HOXC11 protein showed characteristic *α* helix signature peaks at 208 nm and 222 nm and in total 52.8% α-helices, 26.5% β-sheets, 1.3% turns and 19.4% random coils (Figure 3B).

### Localization of HOXC11 protein

**Figure 4:**
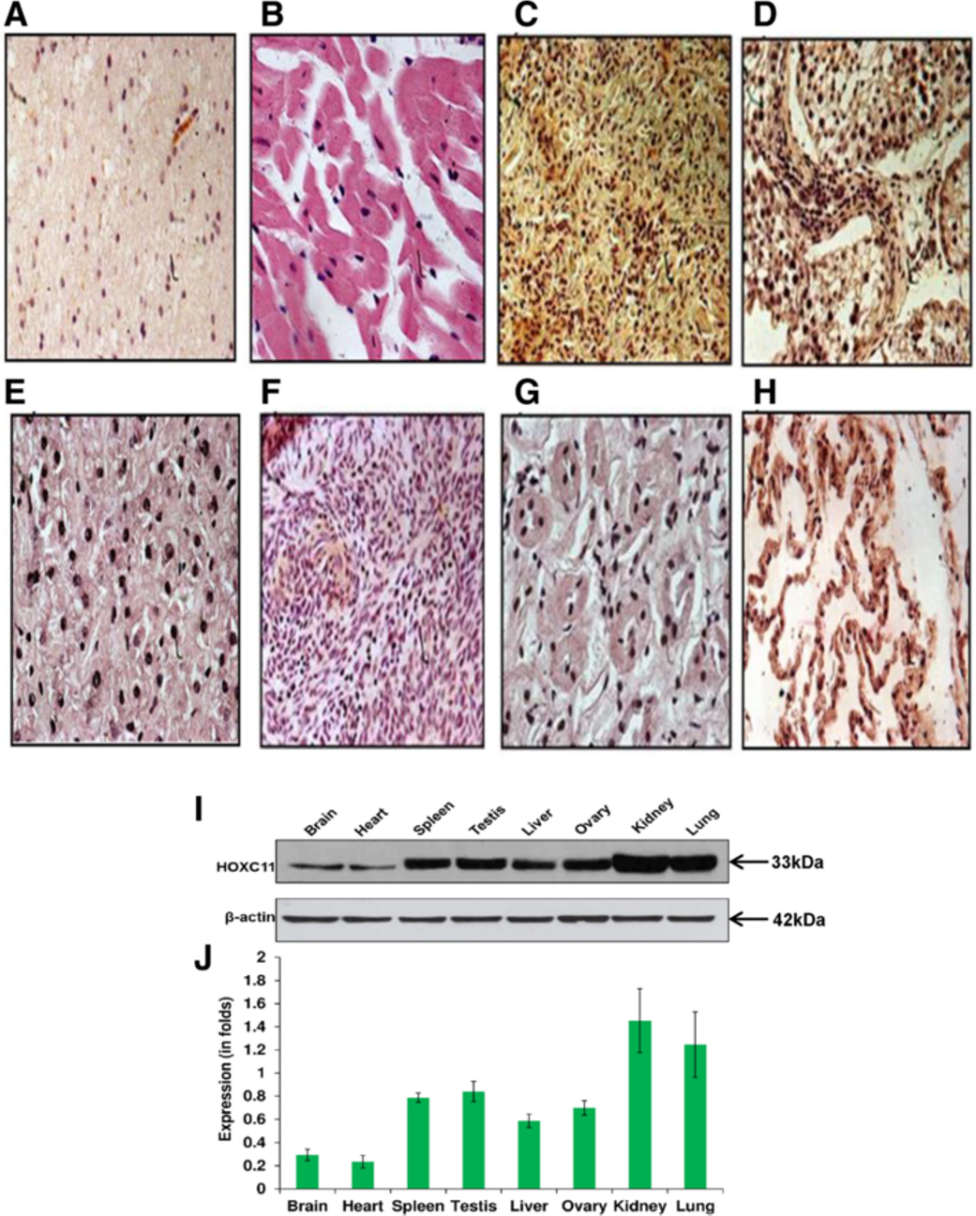
**Localization of HOXC11 protein across the tissues of buffalo.** HOXC11 was detected in the nuclei by IHC using rabbit anti-HOXC11 antibodies and goat anti-rabbit HRP antibodies. Nuclei were counterstained by hematoxylin. Images were taken in bright field (DIC) at 60× magnification. **(A-H)** indicate brain, heart, spleen, testis, liver, ovary, kidney and lung, respectively. **I)** Western blot analysis of HOXC11 protein in different somatic and gonadal tissues of buffalo using rabbit anti-HOXC11 IgG. The differential expression of HOXC11 protein across the tissues was normalized against β-actin. **J)** The expression (in folds) of HOXC11 protein was calculated using Image J software. The standard bars indicate the reproducibility of the experiment.

HOXC11 protein was localized in the nuclei across the tissues of buffalo employing indirect immunohistochemistry assay (Figure 4A-H). The expression of HOXC11 in gonadal and different somatic tissues of water buffalo was studied by immunoblotting using specific antibodies which showed maximum expression in kidney followed by lung and spleen (Figure 4I). The expression of HOXC11 protein across the tissues was normalized using β-actin (Figure 4J).

### HOXC11 interacts with CE-LPH1 element of Lactase-phlorizin hydrolase (LPH) gene promoter

**Figure 5:**
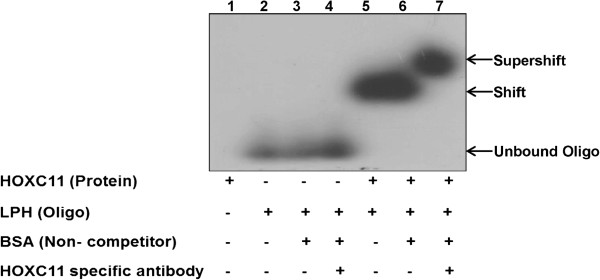
**Electrophoretic mobility shift assay.** DNA binding property of HOXC11 protein deduced by EMSA. α-^32^P labelled double stranded oligos were incubated with refolded HOXC11 protein. The Protein-DNA complex was separated through native polyacrylamide gel. Note the migration of the free and bound α-^32^P labelled double stranded oligos in lanes 1, 2, 3 and 7, respectively.

EMSA was performed with recombinant HOXC11 protein to assess the protein interaction with LPH promoter. The binding of HOXC11 with LPH promoter in the presence of specific anti-HOXC11 antibodies resulted in reduced mobility of the protein-DNA complex. Consequently, a supershift was seen (Figure 5). Whereas, BSA used as a non-competitor for LPH failed to form such complex and therefore no shift was observed. Thus, the data confirms strong interaction of buffalo HOXC11 protein with the LPH promoter.

### Phylogenetic status and domain organization of HOXC11

**Figure 6:**
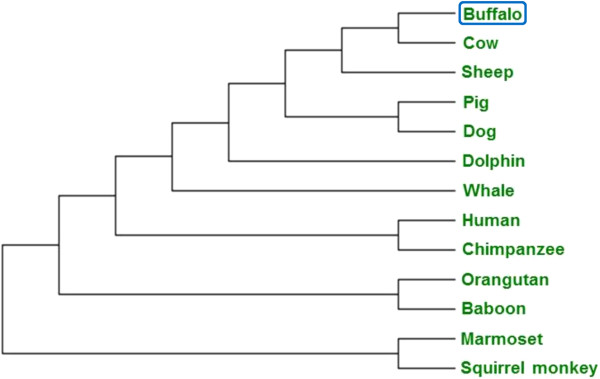
**Phylogenetic status of HOXC11 protein across different species.** The tree construction was based on the amino acid sequences using MEGA 5.2 software employing Maximum Parisomy with Subtree-Prunning- Regrafting (SPR) algorithm.

**Figure 7:**
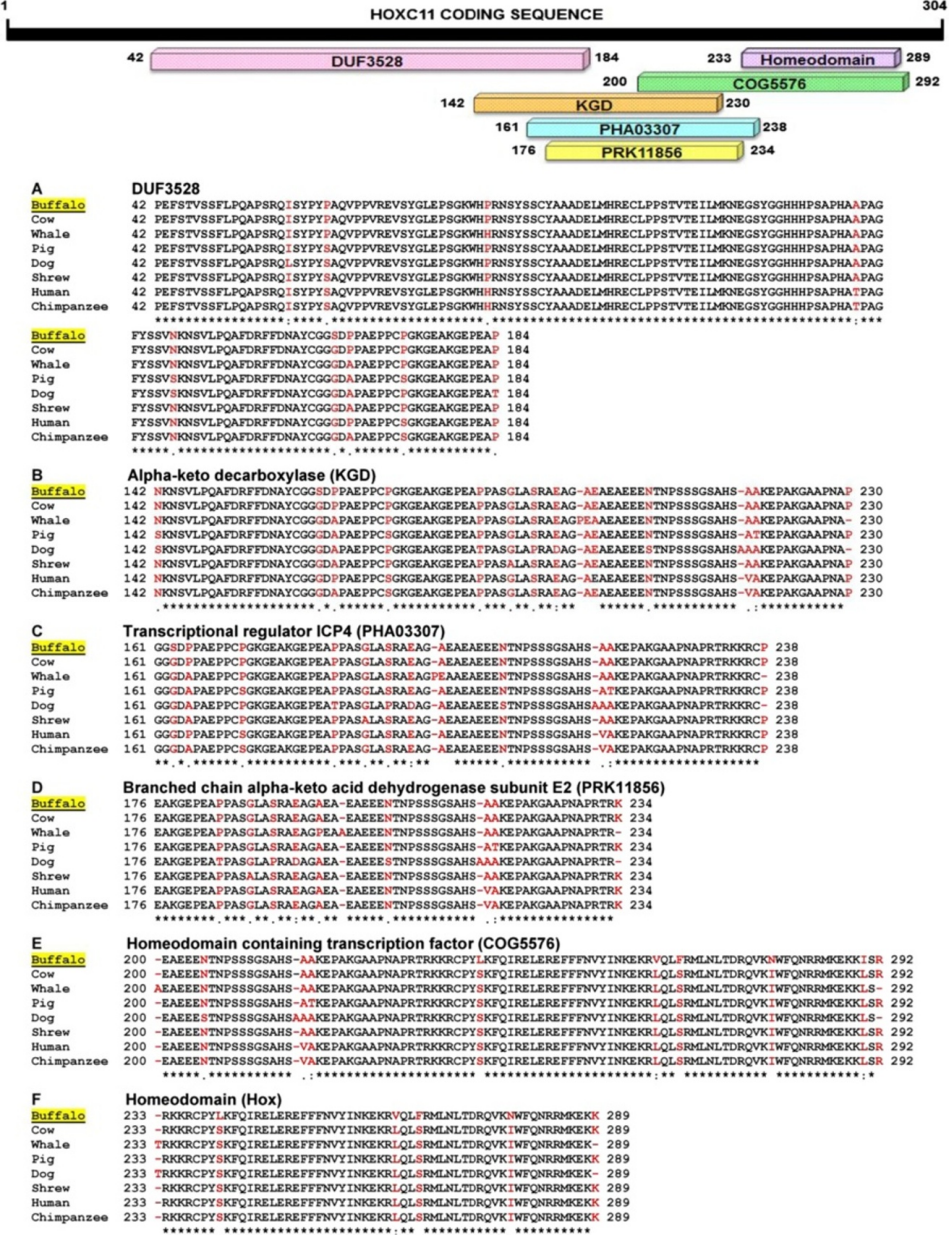
**Domain organization of HOXC11.** The sequences were aligned across the species for six domains namely DUF3528, Alpha-keto decarboxylase, Transcriptional regulator ICP4, Branched chain alpha-keto acid dehydrogenase subunit 2, Homeodomain containing transcription factor and Homeodomain. **(A-F)** represent the amino acid sequences based alignment from different mammalian species. The variations between the sequences have been highlighted in red. Conserved amino acids are indicated by asterisks (*) below.

Multiple sequence alignment showed more than 89.38% homology of buffalo HOXC11 amino acids with that of 17 different species (Additional file 2). Phylogenetic tree constructed using HOXC11 amino acid sequence showed the evolutionary status of different species placing buffalo closer to cattle (Figure 6). *In silico* studies uncovered six putative conserved domains in HOXC11 protein namely DUF3528, alpha-keto decarboxylase (KGD), transcriptional regulator ICP4 (PHA03307), branched chain alpha-keto acid dehydrogenase subunit E2 (PRK11856), homeodomain containing transcription factor (COG5576) and homeodomain all showing high degree of homology across the species (Figure 7).

### 3D structure of HOXC11

**Figure 8:**
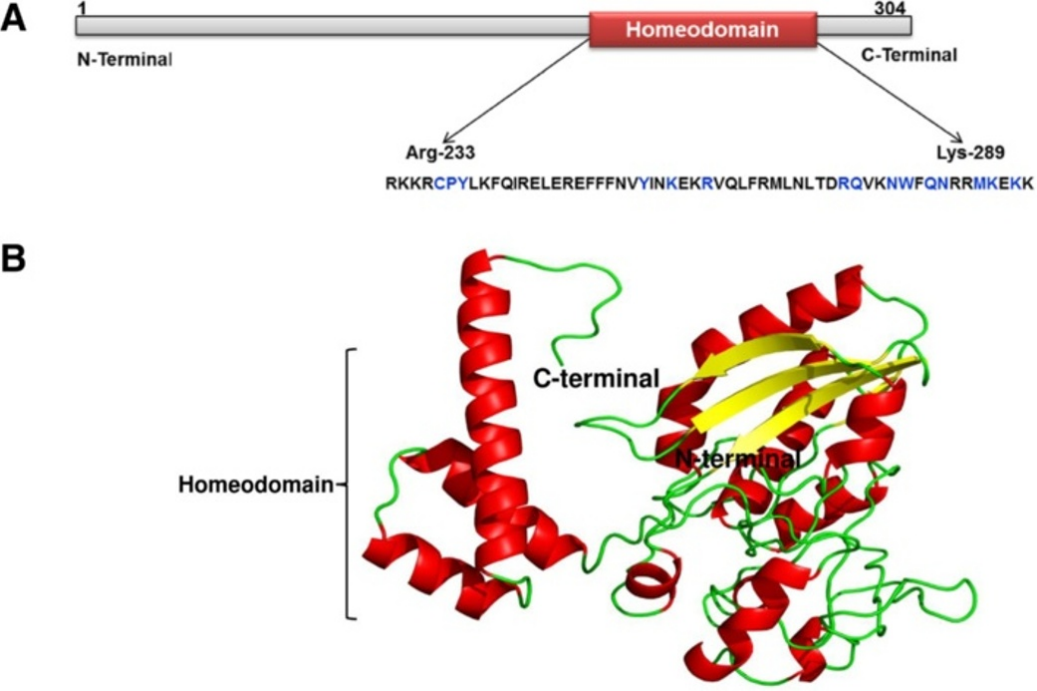
**Modeled 3D structure of the buffalo HOXC11 protein. A)** Diagrammatical representation of homeodomain (56 aa long) and possible binding site residues (in blue) of HOXC11 protein. **B)** The 3D structure of buffalo HOXC11 was generated by I-TASSER and visualized by PyMol (version 1.7). Helix, beta sheets and coils/loops are shown in red, yellow and green, respectively.

Tertiary structure of HOXC11 was generated using the I-TASSER server. The predicted model had a confidence score (C-score) of -3.48 with TM score and RMSD values of 0.33 ± 0.11 and 14.7 ± 3.6 Å, respectively. The HOXC11 protein model was submitted to Protein Model DataBase for which PMDB ID: PM0079553 was assigned. The 3D structure of HOXC11 revealed fifteen possible DNA interacting residues within the homeodomain namely Cys 237; Pro 238; Tyr 239, 256; Lys 259, 286, 288; Arg 262, 274; Glu 275, 289; Asp 278, 282; Trp 279 and Met 285 (Figure 8). Ramachandran plot showed the presence of 95.3% residues in the allowed regions and only 2.4% residues in disallowed regions. The ERRAT 2 score of 82.759 verifies the quality of the model (Additional file 3). The statistical data confirmed that the quality of model is acceptable.

## Discussion

*Hox* genes are found in majority of the species owing to their ardent requirement for pattern formation [21, 22]. HOXC11 protein isolated and purified herein emitted tryptophan spectra at 330 nm revealing the *hitherto* buried nature of tryptophan residues and their presence in the non-polar environment [23]. Further, *in silico* analysis and CD spectra indicated the predominance of α helices encompassing 52.8% of the total HOXC11 protein. The expression of HOXC11 protein has been reported in mouse embryonic tissues like kidney, prostatic urethra, femur, pelvis and gut [6, 24]. HOXC11 protein was localized earlier in the nucleus of T-cell acute lymphoblastic leukemia [4]. We localized the HOXC11 protein in the nuclei of gonads and different somatic tissues of buffalo employing immunohistochemistry approach. Subsequent western blot analysis showed the highest expression of HOXC11 protein in kidney, followed by lung and spleen. Based on the high expression of HOXC11, we construe its involvement in renal, respiratory and immunological activities.

The lactase-phlorizin hydrolase (*LPH*) gene is known to express in the enterocytes of the small intestine [25]. Studies on immunoprecipated human lactase have demonstrated the enzymatic activities against lactose, phlorizin and several glycopeptides. Thus, it is reasonable to expect that the mechanism of this genetic switch is common to all the species. In rats and rabbits, lactase activity is reportedly higher during the neonatal stages which, gradually decreases towards the adulthood [25–27]. Several reports have indicated the down-regulation of *LPH* activity in mammals at the transcriptional level, however, subsequent post-transcriptional mechanisms may also play modifying roles [28–30]. The binding of Cdx-2 (homeodomain protein) with LPH elements in Caco-2 cells has been reported [31]. In the present study, electrophoretic mobility shift assay (EMSA) showed a similar binding affinity of buffalo HOXC11 protein with the promoter element of *LPH* gene. This corroborates the earlier work highlighting potential role in regulating the activation of *LPH* [21] and thereby its intestinal functions in adult buffalo.

Predicting the role of orthologues separated over a long span of time has been a difficult proposition, especially in the context of the multidomain proteins with frequent insertions/deletions. Accordingly, the biological functions of six domains of HOXC11 protein such as DUF3528, Homeodomain, COG, PHA, KGD and PRK are still unclear. *In silico* analysis of HOXC11 amino acid sequence showed the presence of DUF3528 and Homeodomain superfamilies. However, function of DUF3528 superfamily is also not yet established. Interestingly, all the subunits showed high degree of conservation across the species conferring intactness of each domain. High degree (>89%) of HOXC11 amino acid sequence conservation across the species indicates more generalized and mandatory biological roles of this protein in higher eukaryotes. Our study seems to be the first attempt in predicting the three dimensional structure of buffalo HOXC11 protein. The HOXC11 transcription factors containing a highly conserved polypeptide segment, the homeodomain, functions by directly binding with the regulatory regions. This in turn initiates the transcription of other developmental genes [9]. Thus, owing to its conservation in human and non-human systems, the fate of HOXC11 protein may be studied in human renal, respiratory and immunological impairments to highlight its clinical relevance.

## Conclusions

Higher expression of HOXC11 protein in kidney and lung reflects its possible tissue specific functions. Similarly, interaction of HOXC11 with lactase-phlorizin hydrolase (LPH) promoter suggests its possible role in intestinal functions. Detection of fifteen interacting residues within the homeodomain of HOXC11 protein enriches its structure-function relationship. Present study opens up newer avenues for gaining deeper understanding on the actions and mechanisms of HOXC11 protein in regulating expression of other genes having possible clinical relevance.

## Methods

### Sample collection

Buffalo tissues namely brain, heart, kidney, liver, lung, spleen, testis and ovary were procured from the local slaughter house. The slaughter house is duly approved and authorized by the Municipal Corporation, New Delhi, India for the exclusive purpose of ‘slaughtering the animals’, intended for the human consumption as permitted in the Bye-Laws. All these samples were procured strictly in accordance with the guidelines of the Institute’s Ethical and Bio-safety committees with their due approvals. Our Institute’s ethical committee is equivalent to the Institutional Animal Care and Use Committee (IACUC).

### Molecular cloning of Hoxc11

*Hoxc11* gene was amplified by end point PCR using testis cDNA and a set of primers (forward 5′CCG*GAATTC*ATGTTTAACTCGGTCAACCT3′ and reverse 5′CCG*CTCGAG*TTACAG CAAAGGATTTCC3′ accommodating sites for *EcoRI* and *XhoI* restriction enzymes (italicized), respectively, based on *Bubalus bubalis Hoxc11* coding sequence (GenBank accession no. KJ959631) and phusion DNA polymerase (NEB, USA). The reaction conditions involved initial denaturation at 95°C for 3 minutes, followed by 35 cycles each with a subsequent denaturation at 94°C for 1 minute, annealing at 58.2°C for 1.5 minutes and extension at 72°C for 2 minutes followed by a final extension at 72°C for 10 minutes. The resultant amplicons were resolved on 1% agarose gel in 1× TAE buffer, sliced and DNA was eluted using gel extraction kit (Qiagen, Germany). The eluted DNA fragments corresponding to *Hoxc11* gene were cloned between *EcoRI* and *XhoI* sites in pET22a vector (Novagen, USA) to produce recombinant HOXC11 with hexahistidine tag and a thrombin cleavage site at N-terminus (His-HOXC11). Positive clones were confirmed by restriction digestion and sequencing following standard protocol [32].

### Expression and purification of HOXC11

Following the successful cloning, the recombinant plasmid (pET22-His-HOXC11) was used for transforming *E. coli* BL21 (DE3) competent cells for protein expression. The cells were grown to mid log phase in Luria-Bertani medium containing 100 μg/ml ampicillin until an OD_600_ of 0.6 at 37°C was achieved. Recombinant protein expression was induced with 1 mM isopropyl β-D-1-thiogalactopyranoside (IPTG) and the cells were further grown for 4 hours at 37°C. The cells were harvested by centrifugation at 6,000xg for 20 minutes, and pellet was suspended in lysis buffer (50 mM Tris, pH 6.5, 100 mM NaCl and 8 M urea) following continuous shaking at 4°C for 4 hours. Supernatant containing HOXC11 protein was loaded on Ni-nitrilotriacetic acid (Ni-NTA) beads (Qiagen, USA) pre-equilibrated with lysis buffer, followed by refolding of protein using gradient removal of urea and eluted with elution buffer (50 mM Tris, pH 6.5 and 100 mM NaCl) containing imidazole. Subsequently, chromatography fractions containing His-HOXC11 were dialyzed against buffer containing 10 mM Tris, pH 6.5.

### SDS-PAGE and Western blotting analysis

SDS-PAGE was performed on the chromatography fractions and protein was visualized by Coomassie Brilliant Blue R-250 (Sigma Aldrich, USA). For western blot analysis, protein was subjected to 15% (w/v) SDS-PAGE and electro transferred onto nitrocellulose membrane (Bio-Rad Laboratories, CA, USA). Membrane was blocked with 5% bovine serum albumin (Sigma Aldrich, USA) in PBST (1× PBS in 0.05% Tween-20) for 2 hours and incubated overnight with diluted anti-His antibodies (Sigma Aldrich, USA) in blocking buffer (1:1000). The blot was washed with PBST and incubated with horseradish peroxidase conjugated anti-rabbit IgG (Sigma Aldrich, USA) diluted in PBST (1:10,000). After subsequent washing, the blot was developed with 1 mg/ml 3,3′-diaminobenzidine (DAB) (Sigma Aldrich, USA) and 1 μl/ml hydrogen peroxide in phosphate buffer saline.

### Mass spectroscopic analysis

Refolded HOXC11 protein was resolved on SDS-PAGE, band corresponding to the desired molecular weight was gel excised and SDS was removed followed by in-gel digestion carried out by trypsin. Peptides were dissolved with 0.1% trifluoroacetic acid in 50% acetonitrile, mixed with MALDI matrix (10 mg/mL of α-cyano-4-hydroxycinnamic acid) and spotted on MALDI target plate. The peptide mass was acquired using 4800 MALDI-TOF/TOF mass spectrometer proteomics analyzer (Applied Biosystems, USA) in positive ion reflectron mode. Peptide matching and protein identification were performed using Mascot peptide mass fingerprint database (Matrix Science Ltd, UK) following standard search parameters.

### UV spectrofluorimetric measurements

Fluorescence measurements were performed using Cary eclipse spectrofluorimeter (Varian, USA). Spectra of purified HOXC11 protein (100 μg/ml) in 10 mM Tris, pH 6.5 was recorded as the average of 3 individual spectral scans. An excitation and emission spectra were recorded at 280 nm and 300 to 400 nm, respectively. Excitation and emission slit widths each was kept at 5 nm.

### Secondary structure analysis

The secondary structure of HOXC11 was investigated by ProFunc server (http://www.ebi.ac.uk/thornton-srv/databases/ProFunc/) and circular dichroism (CD) polarimeter. For CD spectral analysis, protein (100 μg/ml) spectra were recorded in the far-UV range (200–250 nm) at 30°C using a J-815 circular dichroism spectropolarimeter (Jasco, Japan). A cell with 1 cm optical path was used to record the spectra at scan speed of 200 nm min^-1^ with sensitivity of 50 mdeg and response time of 1 second. The sample compartment was purged with nitrogen, and spectra were averaged over 3 scans. The results are presented as mean residue ellipticity (MRE) [33]. Calculation of secondary structural elements was performed using the CDNN program.

### Generation of antiserum against HOXC11

Animals were procured from the Animal House facility of the National Institute of Immunology, New Delhi, India. Two 8-weeks-old New Zealand white rabbits were immunized by subcutaneous route with 0.59 mg/ml of commercially synthesized peptide (GL Biochem (Shanghai) Ltd., China). Sixteen amino acids long peptide (KEKKISRDRLQYFSGN) was emulsified in complete Freund’s adjuvant. The rabbits were immunized with HOXC11 peptide formulated in incomplete Freund’s adjuvant subsequently blood was collected and sera were isolated. These were used for immunoassays and western blot analysis.

### Enzyme linked immunosorbent assay (ELISA)

Rabbit sera were used to detect recombinant HOXC11 protein by ELISA. Briefly, wells were coated with 100 μl of different dilutions of HOXC11 protein and blocked with 1% BSA in phosphate-buffer saline (blocking buffer). Antigen coated wells were incubated with 100 μl of rabbit anti sera diluted (1:1000) in blocking buffer. After washing with PBST, wells were incubated with 100 μl of horseradish peroxidase labeled anti-rabbit antibody (1:10,000) (Sigma Aldrich, USA) for 1 hour at 37°C. The enzyme reaction was developed by 0.2% o-phenylenediamindihydrochloride (OPD) as the chromogen and hydrogen peroxide (30%) as the substrate. The reaction was terminated by addition of sulfuric acid (5 N) and OD at 492 nm was recorded using an ELISA microplate reader (HT2 Antos HILL, USA). Buffer for dissolving protein (10 mM Tris, pH 6.5) was used as control. Immunoreactivity of the HOXC11 protein, expressed as absorbance values was determined by comparison with the values of control.

### Indirect immunohistochemistry

Paraffin blocks were prepared by immersing tissue samples in neutral buffer containing 10% formalin for 8 hours. Tissues were dehydrated in ascending grade of ethanol, infiltrated and embedded in low melting paraffin at 56°C in a heated oven. The tissue paraffin mold was solidified on a cold plate to form block. Fixed tissues were sectioned (5 μM) using a microtome (MRS3500 Histoline Laboratories, Italy). Tissue sections were deparaffinized and rehydrated in descending grades of ethanol following standard protocols [34]. Endogenous tissue peroxidase was blocked by incubating the sections with hydrogen peroxide solution. Sections were treated with 1% Triton X-100 followed by blocking with 1% bovine serum albumin (BSA). Rabbit anti-HOXC11 sera (1:100) were added to each tissue section and incubated overnight at 4°C in humidified chamber. The appropriate HRP conjugated secondary antibody (1:1000) and diaminobenzidine (DAB) chromogen substrate were used to detect the binding of primary antibodies [34]. Screening was done under differential interference contrast (DIC) using Nikon Eclipse T2 Microscope.

### Protein isolation from the tissues of buffalo

Total cellular extracts of somatic and gonadal tissues were prepared by homogenizing the tissues in RIPA buffer (50 mM Tris, pH 7.6, 150 mM sodium chloride, 1% Na-deoxycholate, 0.1% SDS and 1% NP-40) followed by a constant rotation for 2 hours at 4°C. The extract was then centrifuged at 10,000 rpm for 15 minutes at 4°C. The supernatant containing the protein was collected. The total protein concentration was determined using Bradford assay (Thermo scientific, USA). The expression of HOXC11 protein across the tissues of buffalo was determined employing western blot analysis as mentioned above using anti-HOXC11 rabbit sera as primary antibody and HRP conjugated anti-rabbit IgG (Sigma Aldrich, USA) as secondary antibody. β-actin was used as loading control. All images were analyzed using Image-J (version 1.44) software.

### Electrophoretic mobility shift assay (EMSA)

A binding assay and native PAGE (5%) experiment were performed as described earlier [35, 36]. In brief, α-^32^P-dCTP (3000 Ci/mmol per reaction) labelled ds oligonucleotides (forward: 5′GATCT*TTTAC*AACCTCAGTTG3′ and reverse: 5′A*AAATG*TTGGAGTCAACCTAG3′) were used as probe. These oligos were designed based on CE-LPH1 element of *LPH* gene promoter {TTTA(T/C), the core sequence is italicized} [24]. The probe was heated for 10 minutes at 70°C, cooled for 5 minutes at room temperature and incubated together with protein in binding buffer (10 mM Tris, pH 7.9, 1 mM DTT, 0.1 mM EDTA, 50 mM KCl and 5% glycerol) for 30 minutes at 4°C. As a non-competitor and negative controls, bovine serum albumin, HOXC11 protein and LPH promoter specific oligos, respectively, were used. The specificity of the reaction was assessed using anti-HOXC11 antibody. Native PAGE was performed in 1× TBE buffer at room temperature for 1 hour at 100 volts. Gel was dried and exposed overnight to X-ray film.

### Multiple sequence alignment and phylogenetic status of HOXC11 protein

Database search was conducted to determine the homology of buffalo HOXC11 amino acid sequence (GenBank accession no. AHI17246.1) with that of other species employing BLASTP program (http://blast.ncbi.nlm.nih.gov/Blast.cgi). Multiple sequence alignment and phylogenetic tree were generated using ClustalW (http://www.ebi.ac.uk/clustalw) and MEGA 5.2 software [37], respectively. Conserved domains of HOXC11 protein were ascertained using structure conserved domain database (CDD) available at NCBI (http://www.ncbi.nlm.nih.gov/srtucutre/cdd/).

### 3D structure of HOXC11

Putative 3D model of this protein was ascertained using web based I-TASSER (iterative threading assembly refinement) server [38] involving threading strategy and generated five models of HOXC11 protein. The best model was selected on the basis of confidence score (C-score), defining the quality of the predicted structure. The selected model was validated by PROCHECK and EERAT 2 tools available online [39, 40] and submitted to the Protein Model DataBase (http://bioinformatics.cineca.it/PMDB/) as mentioned earlier.

## Electronic supplementary material

Additional file 1: **MALDI-peptide mass fingerprints of HOXC11 protein.** A) The MS spectra of HOXC11 depicting the peaks corresponding to tryptic digested protein fragments (peptides). B) Mascot based identification of HOXC11 protein. Details of the MALDI generated peaks corresponding to their m/z values searched against the database. (PPTX 111 KB)

Additional file 2: **Multiple sequence alignment of** ***Bubalus bubalis*** **HOXC11 protein across the species.** *In silico* analysis confirmed more than 89% homology of *Bubalus bubalis* HOXC11 protein with that of other species. Identical amino acids amongst the species are indicated by an asterisk (*). (PPTX 788 KB)

Additional file 3: **Validation of 3D structure of HOXC11 protein.** A) Ramachandran Plot for buffalo HOXC11 protein. B) The ERRAT 2 score (82.759) calculated as a quality factor of predicted model. ERRAT 2 score higher than 50 signifies the good quality of the model. (PPTX 488 KB)
