# Supplementary figures and images for: Differential expression of Homeobox C11 protein in water buffalo Bubalus bubalis and its putative 3D structure
Source: BMC Genomics. 2014 Jul 30;15(1):638. doi: 10.1186/1471-2164-15-638 (PMC4139611; doi:10.1186/1471-2164-15-638)

## Slide 1
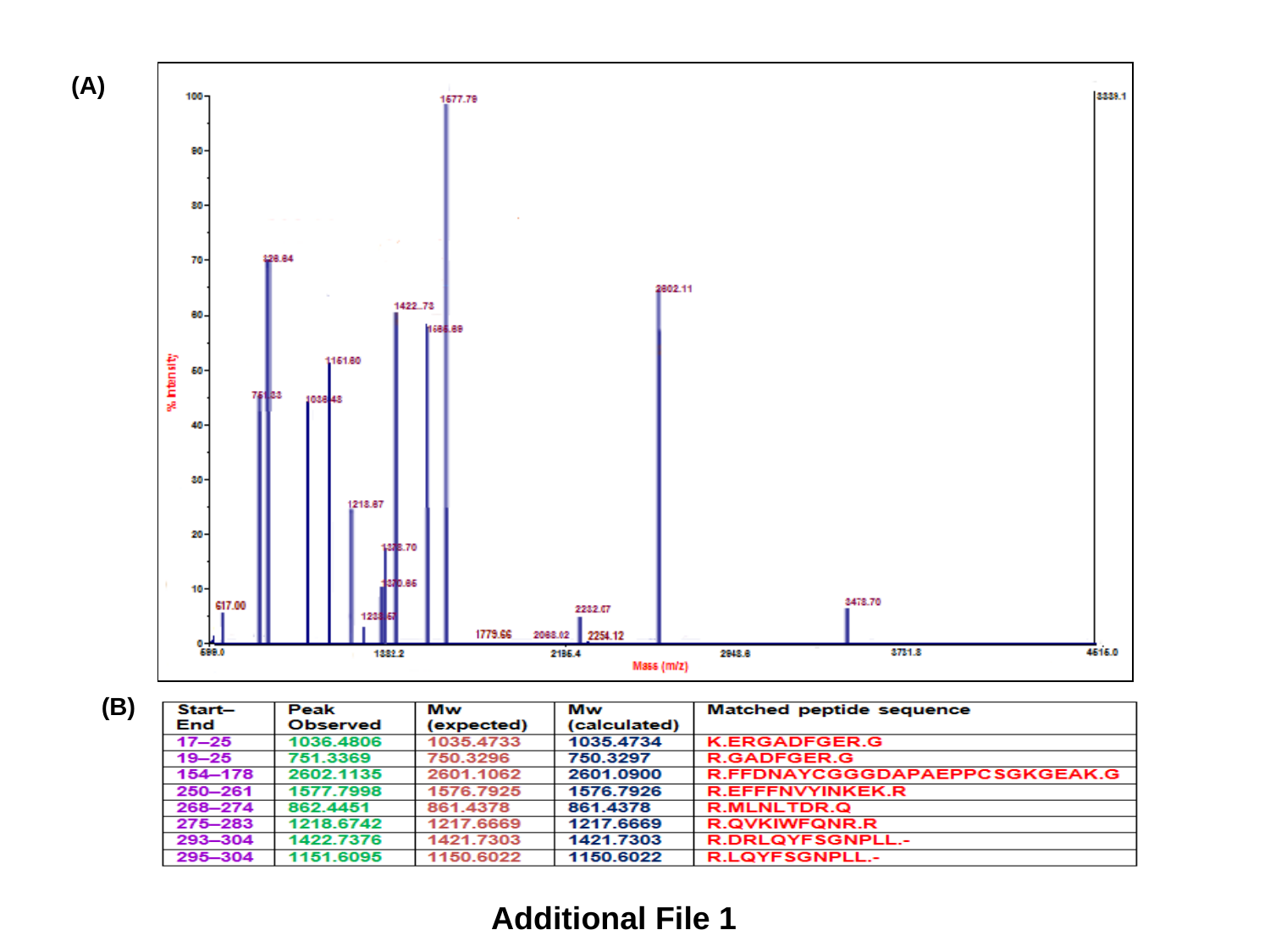

(A)
(B)
Additional File 1

Supplement: Supplementary file 1 — Additional file 1: MALDI-peptide mass fingerprints of HOXC11 protein. A) The MS spectra of HOXC11 depicting the peaks corresponding to tryptic digested protein fragments (peptides). B) Mascot based identification of HOXC11 protein. Details of the MALDI generated peaks corresponding to their m/z values searched against the database. (PPTX 111 KB) [file 12864_2014_6361_MOESM1_ESM.pptx]

## Slide 1
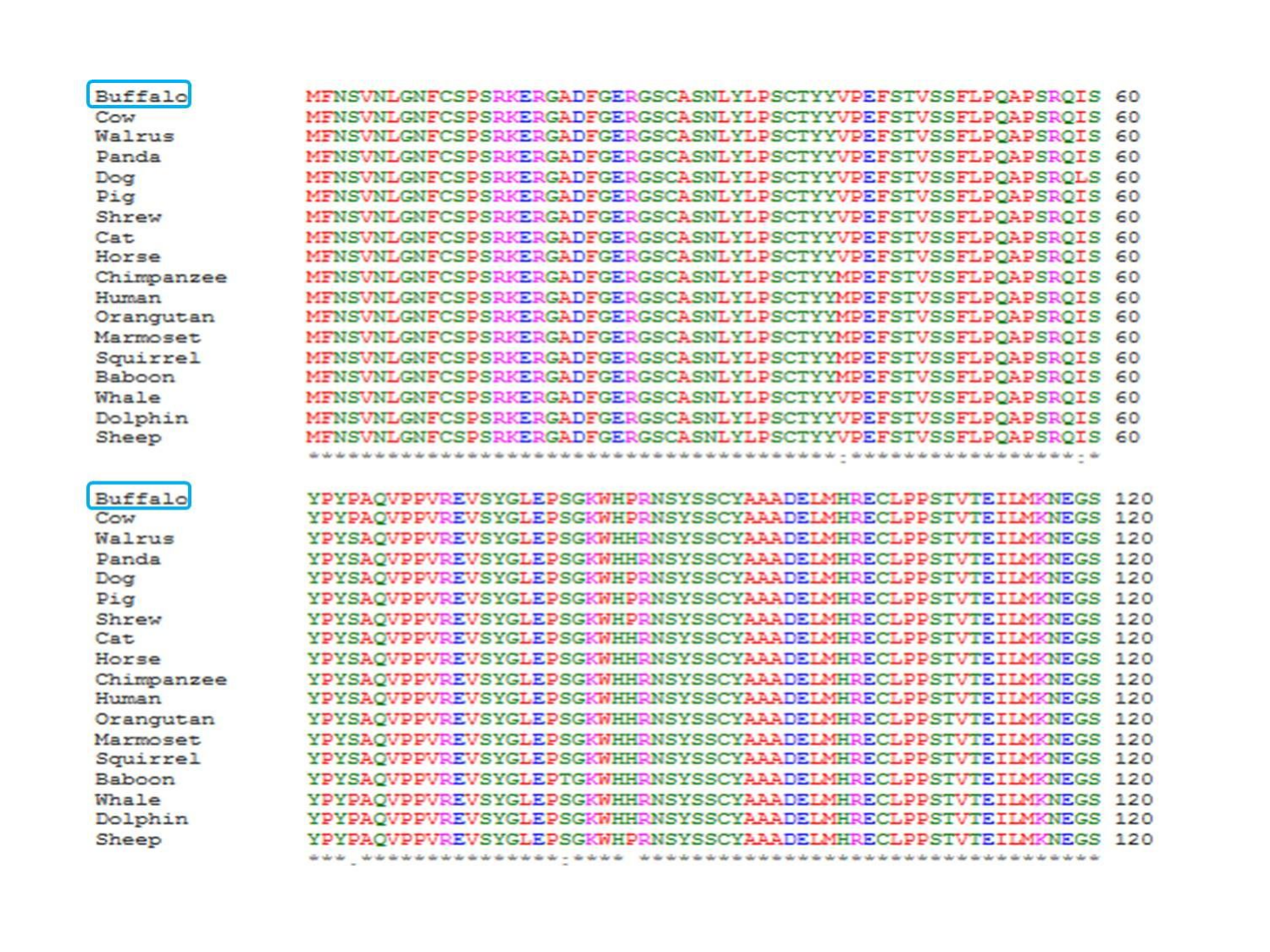

## Slide 2
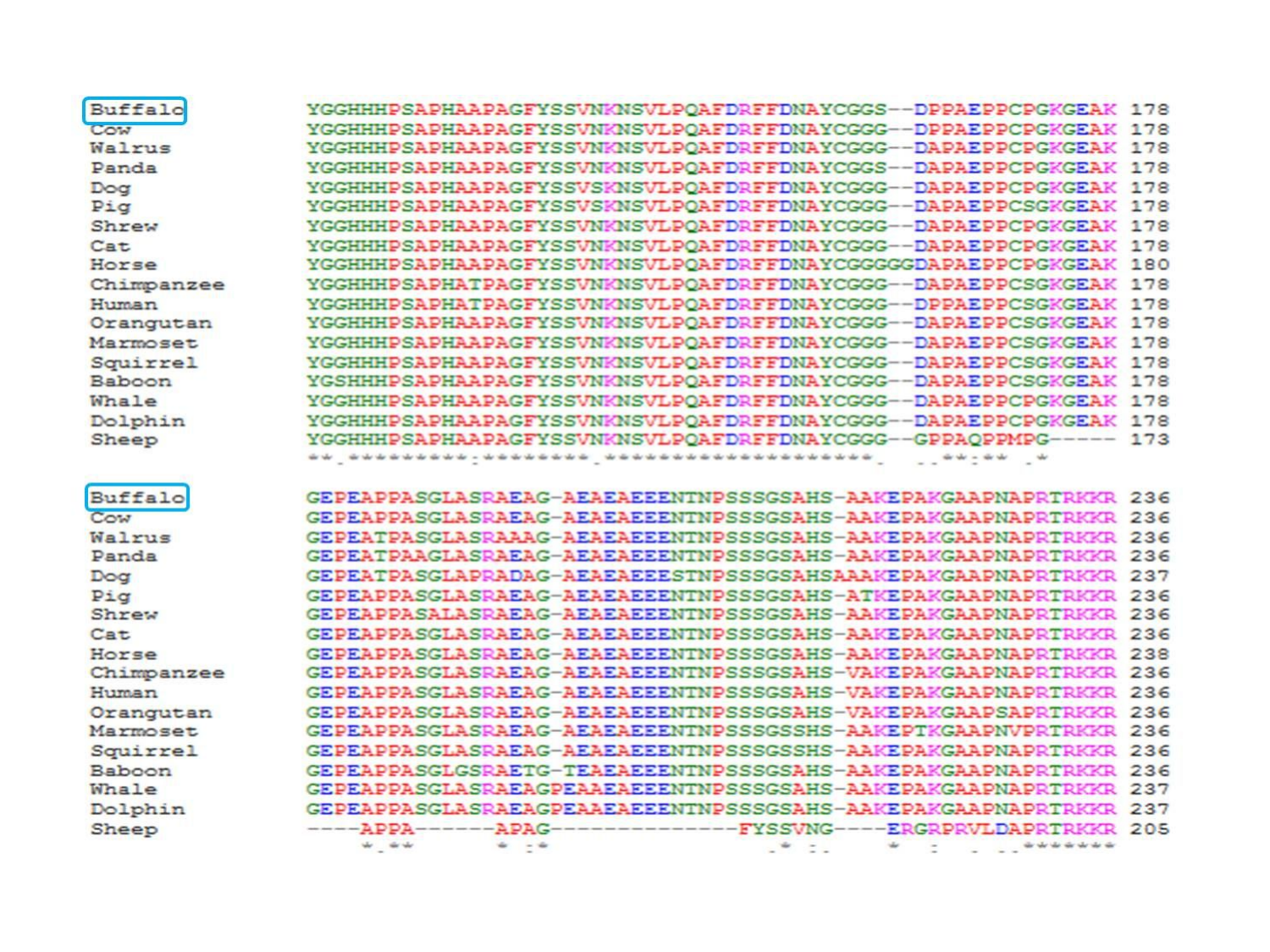

## Slide 3
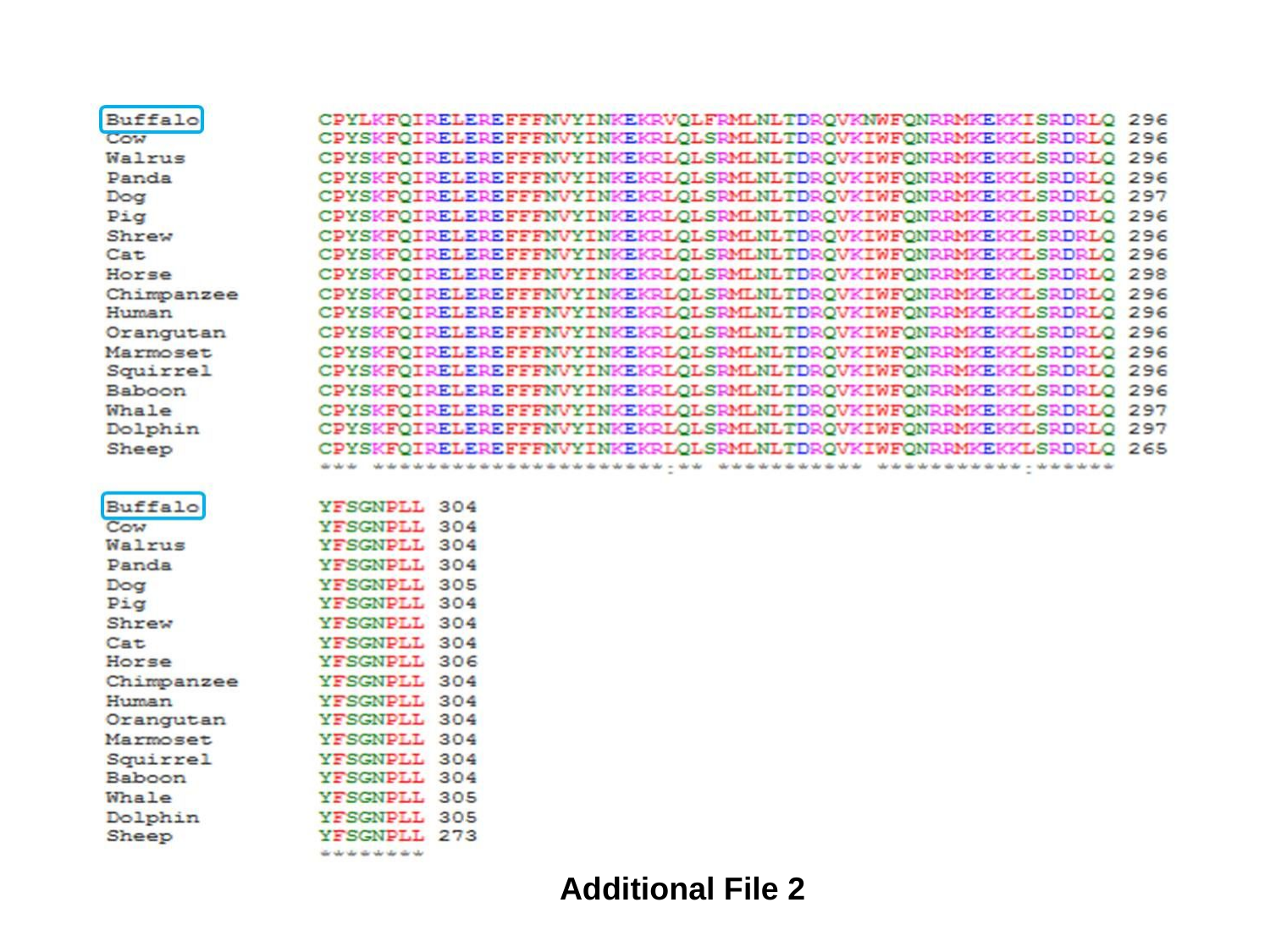

Additional File 2

Supplement: Supplementary file 2 — Additional file 2: Multiple sequence alignment of Bubalus bubalis HOXC11 protein across the species. In silico analysis confirmed more than 89% homology of Bubalus bubalis HOXC11 protein with that of other species. Identical amino acids amongst the species are indicated by an asterisk (*). (PPTX 788 KB) [file 12864_2014_6361_MOESM2_ESM.pptx]

## Slide 1
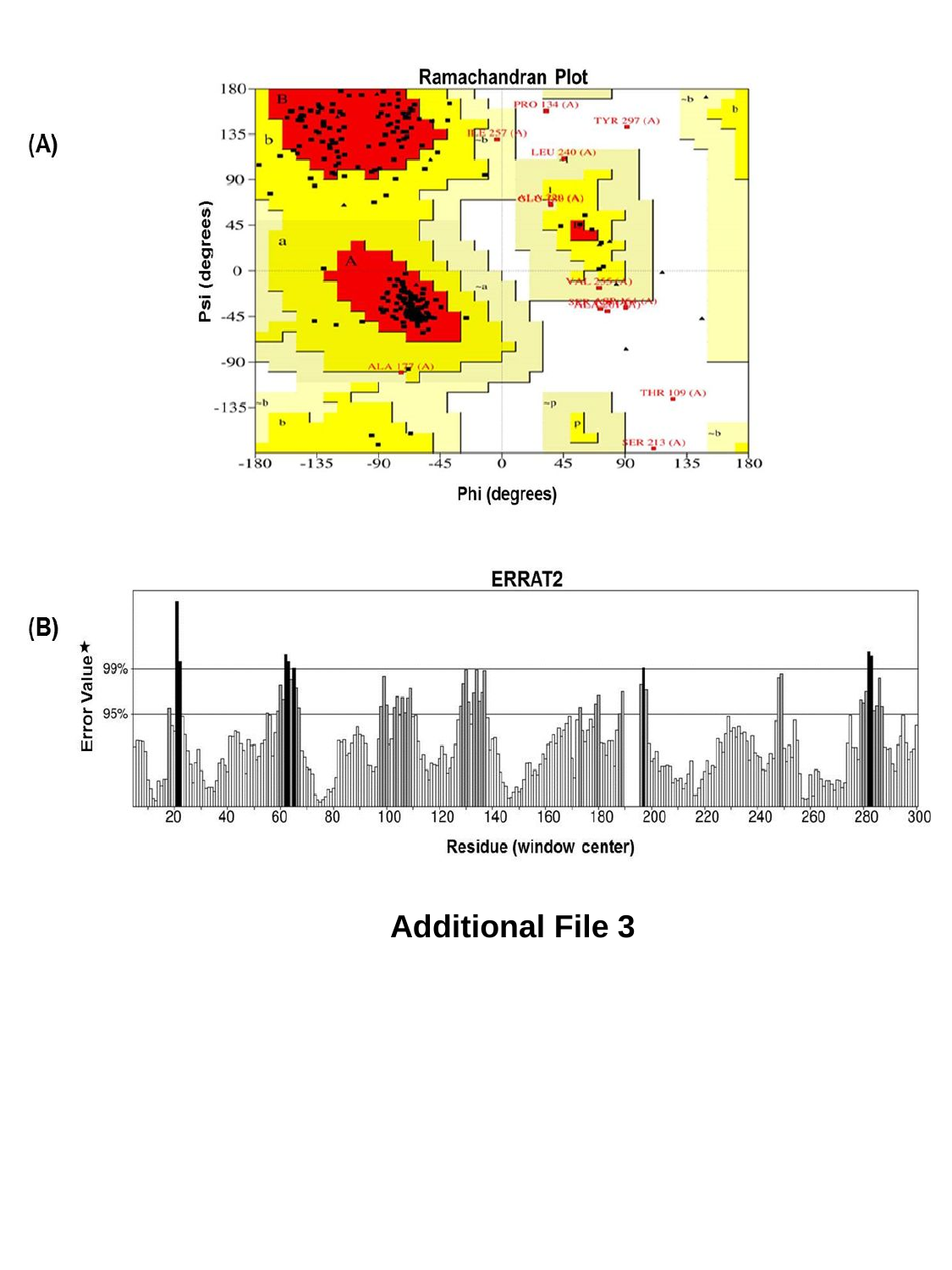

Additional File 3

Supplement: Supplementary file 3 — Additional file 3: Validation of 3D structure of HOXC11 protein. A) Ramachandran Plot for buffalo HOXC11 protein. B) The ERRAT 2 score (82.759) calculated as a quality factor of predicted model. ERRAT 2 score higher than 50 signifies the good quality of the model. (PPTX 488 KB) [file 12864_2014_6361_MOESM3_ESM.pptx]
